# α-Lipoic acid mitigates age-related macular degeneration via ferroptosis: integrative multi-omics and network pharmacology

**DOI:** 10.3389/fphar.2025.1626907

**Published:** 2025-07-31

**Authors:** Yaqiong Zhang, Yuanyuan Chen, Chenglin Sun, Fang Li, Yin Shen

**Affiliations:** ^1^Eye Center, Renmin Hospital of Wuhan University, Wuhan, Hubei, China; ^2^Xiangyang No.1 People’s Hospital, Hubei University of Medicine, Xiangyang, Hubei, China; ^3^Department of Ophthalmology, Zhongnan Hospital of Wuhan University, Wuhan, Hubei, China; ^4^Frontier Science Center for Immunology and Metabolism, Medical Research Institute, Wuhan University, Wuhan, Hubei, China

**Keywords:** age-related macular degeneration (AMD), ferroptosis, α-Lipoic acid (ALA), network pharmacology, multi-omics

## Abstract

**Background:**

Age-related macular degeneration (AMD) is a leading cause of irreversible vision loss among the elderly. α-Lipoic acid (ALA), a naturally occurring antioxidant and iron-chelator, has shown potential in modulating ferroptosis, but its mechanism in AMD remains unclear.

**Methods:**

Network pharmacology, transcriptomic profiling, and machine learning were used to identify potential molecular targets of ALA in AMD. Core genes were identified through interaction network construction, functional enrichment analysis, and machine learning-based screening. Molecular docking and molecular dynamics simulations were performed to assess the binding affinity and stability between ALA and its predicted targets. *In vivo* validation was conducted using a sodium iodate (SI)-induced AMD mouse model, with retinal structure, function, oxidative stress, and gene expression evaluated through behavioral tests, histological staining, and qRT-PCR.

**Results:**

We identified six ferroptosis-related core targets (*AHCY, DHODH, MAPK1, MAPK8, NOS2,* and *HMOX1*) of ALA implicated in AMD. Molecular docking revealed strong binding affinities between ALA and these six targets, with dynamic simulations confirming stable interactions, particularly with *HMOX1* and *MAPK1*. In the SI-induced AMD mouse model, ALA significantly preserved retinal structure, maintained visual function, and reduced oxidative stress and iron accumulation. qRT-PCR confirmed that ALA exerted differential effects on the expression of the six genes, demonstrating a context-dependent regulatory mechanism.

**Conclusion:**

This study provides multi-level evidence that ALA protects against AMD by modulating ferroptosis-related pathways and restoring retinal structural integrity and functions. These findings warrant further investigation into the therapeutic potential of ALA in AMD.

## 1 Introduction

Age-related macular degeneration (AMD) is a progressive retinal disorder and a leading cause of irreversible vision loss among the elderly worldwide (Guymer and Campbell, 2023). It is clinically characterized by central visual impairment, metamorphopsia, and difficulty with tasks requiring high visual acuity, such as reading or facial recognition (Flores et al., 2021; Guymer and Campbell, 2023; Stahl, 2020). Pathologically, AMD primarily affects the macula through the dysfunction and loss of retinal pigment epithelium (RPE) cells (Kaarniranta et al., 2020), secondary photoreceptor degeneration (Pfau et al., 2020), and disruption of Bruch’s membrane (Kumaramanickavel, 2016). Although the precise pathogenesis of AMD remains incompletely understood, it is known to involve a complex interplay of oxidative stress, lipid accumulation, neovascularization, chronic inflammation, and degeneration of the RPE, ultimately leading to photoreceptor dysfunction and macular atrophy (Amini et al., 2023; Datta et al., 2017; Fleckenstein et al., 2021; Zhou et al., 2024). AMD is believed to cause ∼9% of all cases of blindness and is estimated to affect 288 million people globally by 2040 (Keenan et al., 2021; Stahl, 2020), underscoring its substantial public health impact. Currently, intravitreal injection of anti-vascular endothelial growth factor (anti-VEGF) represents the standard treatment for neovascular AMD (Cheung et al., 2017); however, its efficacy is limited in non-exudative (dry) forms (Śpiewak et al., 2024), particularly in geographic atrophy, the treatment options for which are highly limited (Wheeler et al., 2024). Moreover, existing treatments predominantly target downstream angiogenic pathways, rather than addressing the upstream pathological mechanisms that initially drive AMD development. Therefore, there is an urgent need to identify novel molecular targets and develop mechanism-based therapies for AMD.

Ferroptosis, a type of programmed cell death, has been recognized as a critical contributor to the development of AMD (Gupta et al., 2023). Ferroptosis is characterized by dysregulated iron metabolism, reactive oxygen species (ROS) accumulation, and glutathione (GSH) depletion, which collectively result in iron-dependent peroxidation of lipids and subsequent disruption to cellular membrane integrity (Jiang et al., 2021). Notably, elevated levels of intracellular iron, lipid peroxides, and reduced antioxidant capacity have been observed in AMD-affected retinal tissues (Zhao et al., 2021), supporting a crucial role for ferroptosis in AMD pathogenesis. In addition, ferroptosis has been implicated in the degeneration of RPE cells (Liu et al., 2024), which are particularly susceptible to oxidative stress and iron overload due to their high metabolic activity and continuous exposure to light. The use of iron chelators, such as deferiprone, has shown protective effects against AMD (Hadziahmetovic et al., 2011; Lukinova et al., 2009; Song et al., 2014). Furthermore, ferroptosis can also enhance chronic inflammation within the retinal microenvironment, contributing to AMD progression (Zhao et al., 2025). Hence, targeting ferroptosis pathways may offer a promising therapeutic option to prevent or mitigate retinal degeneration in AMD.

α-Lipoic acid (ALA) is a naturally occurring organosulfur compound that functions as a potent antioxidant and modulator of cellular redox balance (Salehi et al., 2019). Several studies suggest that ALA may have therapeutic potential in AMD treatment. A randomized controlled trial reported that ALA supplementation improved visual function and quality of life in patients with dry AMD (Tao et al., 2016), likely by enhancing retinal antioxidant defenses (Sun et al., 2012). ALA has also been proposed as a potential therapy for geographic atrophy, the advanced form of dry AMD, although clinical outcomes remain inconclusive (Kim et al., 2020). In a light-induced retinal degeneration mouse model, ALA demonstrated protective effects attributed to its combined antioxidant and iron-chelating properties (Zhao et al., 2014). Specifically, ALA attenuated oxidative damage and preserved photoreceptor integrity by downregulating genes associated with oxidative stress, inflammation, and iron accumulation. These findings suggest that ALA may exert protective effects in AMD by modulating ferroptosis-related pathways, which remain to be fully elucidated.

In this study, we systematically explore the role of ALA in modulating ferroptosis-associated pathways in AMD. We employed an integrated approach combining network pharmacology, transcriptomic analysis, and machine learning to identify potential molecular targets of ALA relevant to AMD and ferroptosis. We also utilized molecular docking and molecular dynamics simulations to assess the binding stability between ALA and its key targets. Finally, we validated the protective effects of ALA in a retinal degeneration mouse model.

## 2 Materials and methods

### 2.1 Target screening

Firstly, the chemical structure of ALA was obtained from the PubChem database, and its potential protein targets were predicted using six databases, including SwissTargetPrediction, PharmMapper, Comparative Toxicogenomics Database (CTD), TargetNet, HIT, and Similarity Ensemble Approach (SEA). Secondly, the genes related to AMD were retrieved from the GeneCards (https://www.genecards.org/), DisGeNET (https://disgenet.com/), and OMIM (https://www.omim.org/) databases using the keyword “age-related macular degeneration.” Ferroptosis-related genes were obtained from the FerrDb V2 database (Zhou et al., 2023). All gene sets were merged and deduplicated to generate a comprehensive list of ALA-ferroptosis-AMD interactions. Additionally, all predicted targets were standardized using UniProt and mapped to official gene symbols via the UniProt ID mapping tool to ensure data consistency (Kim et al., 2021). The intersection of ALA-associated targets with AMD- and ferroptosis-related genes was visualized using a Venn diagram, and overlapping genes were retained as candidate targets for further analysis.

### 2.2 Network pharmacology and enrichment analysis

To explore the interactions between ALA and AMD- or ferroptosis-related genes, we constructed an ALA-ferroptosis-AMD network using Cytoscape (version 3.9.1) (Shannon et al., 2003), with nodes representing ALA, the ferroptosis-related genes, and AMD-related genes, while edges indicated predicted associations.

The protein-protein interaction (PPI) network was established using the STRING database with a minimum interaction score threshold of 0.4 (Szklarczyk et al., 2019). The key genes were identified using the CytoHubba plugin based on topological parameters such as degree centrality (Chin et al., 2014). Gene Ontology (GO) and Kyoto Encyclopedia of Genes and Genomes (KEGG) enrichment analyses were performed using the clusterProfiler package in R (v4.2.2) (Wu M. et al., 2021). Pathways with a false discovery rate (FDR) < 0.05 were considered statistically significant. Enrichment results were visualized as bubble plots using the ggplot2 package in R.

### 2.3 Transcriptomic integration

Transcriptomic data related to human AMD was retrieved from the GEO database (GSE135092), comprising 104 AMD samples and 433 controls. Probe annotation and gene symbol conversion were performed using custom Perl scripts. The raw expression data were then subjected to log2 transformation and quantile normalization. To remove potential batch effects, the ComBat algorithm from the sva package was applied, yielding a standardized expression matrix for downstream analysis. The differentially expressed genes (DEGs) between AMD and control samples were identified using the limma package (Ritchie et al., 2015). Genes with a fold change greater than 2 (|log_2_FC| > 1) and an adjusted *p*-value <0.05 were considered statistically significant. Overlapping genes between the DEGs and previously identified ALA-ferroptosis-AMD targets were defined as significant differential core genes (SDECGs) and visualized using boxplots and heatmaps. The chromosomal localizations of these SDECGs were extracted using Perl scripts, and circular diagrams were generated using the RCircos package. Pearson correlation analysis (r > 0.6) was performed to evaluate the co-expression relationships among SDECGs, and gene-gene interaction networks were constructed accordingly.

### 2.4 Immune cell infiltration and immune signature analysis

CIBERSORT and single-sample gene set enrichment analysis (ssGSEA) were performed on the transcriptomic dataset to assess the immune landscape associated with AMD and its potential modulation by ALA-related targets (Chen et al., 2018; Subramanian et al., 2005). Specifically, CIBERSORT was run 1,000 times on the samples to estimate the relative abundance of 22 immune cell types in each sample. The ssGSEA scores were calculated with predefined immune-related gene sets, representing the enrichment level of immune cell signatures or pathways across individual samples. Subsequently, differential analysis of immune cell infiltration between the AMD and control groups was performed using the Wilcoxon rank-sum test. Correlation analysis was performed between the previously identified SDECGs and ssGSEA scores to identify immune-gene associations. The correlation results were visualized using the ComplexHeatmap package.

### 2.5 Machine learning

Two machine learning algorithms, including Least Absolute Shrinkage and Selection Operator (LASSO) regression and Random Forest (RF), were used to identify key ferroptosis-related genes. LASSO regression was applied with 10-fold cross-validation to identify the optimal penalty parameter (λ = 0.01), resulting in a subset of genes with non-zero coefficients. In parallel, the RF algorithm constructed 500 decision trees and ranked gene features based on their Gini importance, selecting those with scores exceeding 9.0. The intersection of both methods was considered high-confidence candidate genes for further validation. A nomogram model was subsequently constructed to visualize and quantify their diagnostic contribution.

### 2.6 Molecular docking and molecular dynamics simulations

The three-dimensional structures of ALA and the key target proteins were retrieved from the PubChem and Protein Data Bank (PDB) databases, respectively. Molecular docking was performed using AutoDock Vina (v1.1.2) to predict binding conformations and calculate binding affinities. Heatmaps of docking scores were generated, and the docking poses were visualized in three dimensions using PyMOL. Subsequently, molecular dynamics (MD) simulations were performed using GROMACS 2022 with the Amber99SB-ILDN force field to assess the stability of the ALA-protein complexes. Each complex was solvated in a cubic TIP3P water box (10 × 10 × 10 nm^3^) with a minimum margin of 1.2 nm from the protein surface. Counterions were added to neutralize the system. Long-range electrostatic interactions were treated using the particle-mesh Ewald (PME) method. The cutoff distances for Coulombic and van der Waals interactions were set to 1.0 nm. Energy minimization was performed using the steepest descent algorithm for 50,000 steps. Equilibration was conducted under the NVT ensemble at 300 K and the NPT ensemble at 1 bar, using the Langevin thermostat and Berendsen barostat, respectively. A 100-nanosecond production run was then performed under periodic boundary conditions.

MD analyses included root mean square deviation (RMSD) to assess global conformational stability, radius of gyration (Rg) to reflect protein compactness, root mean square fluctuation (RMSF) to evaluate residue flexibility, and solvent-accessible surface area (SASA) to measure solvent exposure. The binding free energy was estimated using the gmx MMPBSA tool, with more negative values indicating stronger affinity. Free energy landscape (FEL) analysis was performed using RMSD and Rg as collective variables, with blue regions indicating the most stable conformational states of the system.

### 2.7 Sodium iodate (SI)-induced retinal degeneration animal model

Male C57BL/6J mice (6–8 weeks old, 20–25 g) were obtained from Beijing Vital River Laboratory Animal Technology Co., Ltd. (Beijing, China) and housed in a pathogen-free facility under standard conditions (12-h light/dark cycle, 22°C ± 2°C, 50%–60% humidity) with free access to food and water. All animal experiments were approved by the Institutional Animal Care and Use Committee of Wuhan University and Hubei Medical University, and performed in accordance with the ARVO Statement for the Use of Animals in Ophthalmic and Vision Research.

Mice were randomly assigned to three groups: control, sodium iodate (SI), and SI + ALA groups (n = 6 per group). Mice in the SI + ALA group received intraperitoneal injections of ALA (10 mg/kg/day; T5625, >99% purity, Sigma-Aldrich, St. Louis, MO, United States) dissolved in sterile PBS for 7 consecutive days. On day 8, SI (25 mg/kg; S4007, Sigma Industries) dissolved in sterile PBS was administered via tail vein injection. Mice in the SI group received PBS for 7 days followed by SI injection, while the control mice received PBS for the first 7 days and on day 8. After SI administration, mice in the SI + ALA group continued to receive ALA (10 mg/kg/day) via intraperitoneal injection for an additional 15 days, whereas mice in the SI and control groups received equivalent volumes of saline.

At the end of the treatment, animals were euthanized, and the eyes were enucleated for downstream analyses. For histological analysis, eyes were fixed in 4% paraformaldehyde (PFA) for 24 h, dehydrated through a graded ethanol series, cleared in xylene, and embedded in paraffin. For cryo-sectioning, retinas were fixed in 4% PFA for 30 min, dehydrated in a sucrose gradient (10%–30%), embedded in optimal cutting temperature (OCT) compound, and cryo-sectioned at 10 μm using a Leica cryostat.

### 2.8 Electroretinography (ERG) and optomotor response (OMR)

Following overnight dark adaptation (12 h), electroretinography (ERG) was performed using the Reti-MINER IV system on day 5, day 10, and day 15 after SI injection (Wu T. et al., 2021). Mice were anesthetized and maintained at 37°C. A reference electrode was inserted subcutaneously into the forehead, a ground electrode was placed at the base of the tail, and a recording electrode was positioned on the cornea using a contact lens. Scotopic ERG responses were recorded at increasing flash intensities ranging from 0.01 to 10 cd·s/m^2^. Visual acuity was assessed using the optomotor response (OMR) test on day 15 after SI injection (Wu, et al., 2021b). Mice were placed on a platform surrounded by a virtual cylinder of rotating vertical gratings (100% contrast, 12°/s). Head-tracking responses to the rotating gratings were recorded across spatial frequencies from 0.042 to 0.336 cycles/degree.

### 2.9 Histological staining and oxidative stress measurement

Paraffin-embedded retinal sections (5–8 μm) were stained with hematoxylin and eosin (H&E) for morphological evaluation. Iron accumulation was assessed using Prussian blue staining with Perl’s reagent (Solution A and B, mixed 1:1) (Wuhan Service Bio-Technology Co. Ltd.) for 1 h at room temperature, followed by nuclear counterstaining. After dehydration, the sections were observed under a light microscope (BX53, Olympus, Japan). Iron-positive areas were quantified using ImageJ software with red particle enhancement as previously described (Zhang et al., 2018). Reactive oxygen species (ROS) levels were evaluated in frozen retinal sections using dihydroethidium (DHE) staining. Sections were incubated with 1 μmol/L DHE at 37°C for 30 min in the dark. Fluorescence intensity was measured under a fluorescence microscope (BX53, Olympus, Japan) and used as an indicator of ROS accumulation.

Retina and serum were obtained from each group of mice, weighed, and centrifuged. Malondialdehyde (MDA) and GSH levels were detected using commercial kits (Beyotime Institue of Biotechnology, Jiangsu, China) following the manufacturer’s instructions. The absorbance at 532 nm and 412 nm (for MDA and GSH, respectively) were measured by a microplate reader (Multiskan GO; Thermo Scientific, MA, United States).

### 2.10 Real-time quantitative PCR (qRT-PCR)

Total RNA was extracted from retinal tissues using TRIzol reagent (Invitrogen, United States) according to the manufacturer’s instructions. Complementary DNA (cDNA) was synthesized using PrimeScript™ RT Master Mix (Takara, Japan) following the recommended protocol. Quantitative real-time PCR (qRT-PCR) was performed in triplicate using SYBR Green Master Mix on a QuantStudio™ 5 Real-Time PCR System (Applied Biosystems, United States). The primer sequences are listed in Supplementary Table S1. GAPDH was used as the internal control, and relative gene expression levels were calculated using the 2^−ΔΔCT^ method. All qPCR experiments were performed in three biologically independent replicates, and four retinal sections were analyzed per replicate.

### 2.11 Statistical analysis

All statistical analyses were performed using GraphPad Prism 9.0 (GraphPad Software, United States) and R software (v4.2.2). Data were expressed as mean ± standard deviation (SD) unless otherwise specified. Comparisons between two groups were conducted using unpaired Student’s *t*-test. One-way analysis of variance (ANOVA) followed by Dunnet’s *post hoc* test was used for comparisons among multiple groups. The Benjamini-Hochberg false discovery rate (FDR) correction method was applied to adjust *p*-values for multiple comparisons. A *p*-value <0.05 was considered statistically significant. All experiments were performed with at least three biological replicates unless otherwise stated.

## 3 Results

### 3.1 Target identification, network construction, and functional enrichment analysis

**FIGURE 1:**
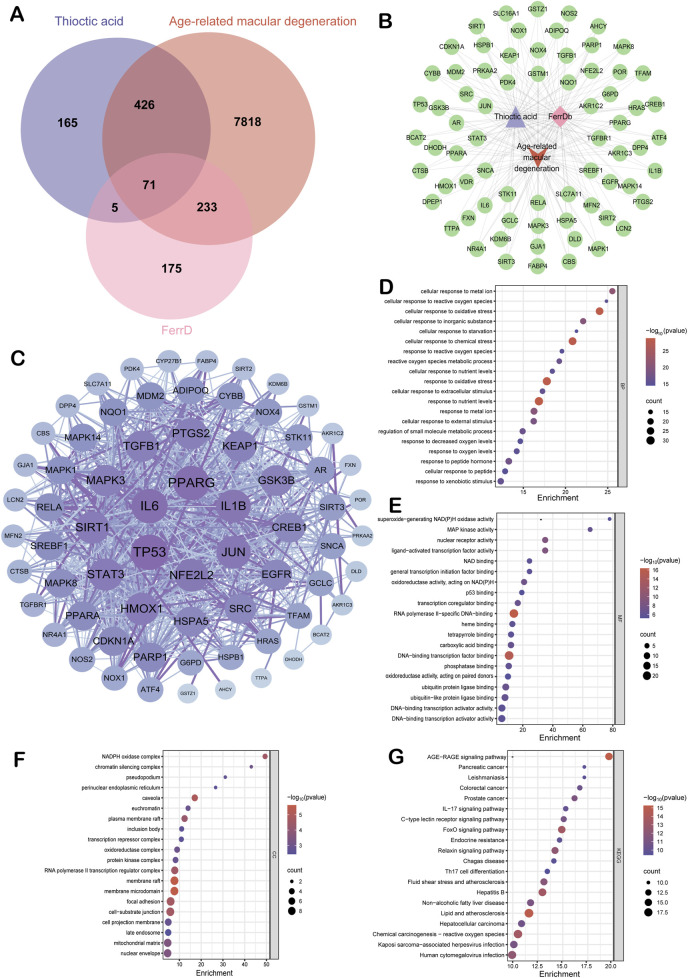
Molecular targets identified by network pharmacology. **(A)** Venn diagram illustrating the intersection among 8,548 AMD-associated genes, 667 ALA-targeted proteins, and 484 ferroptosis regulators. **(B)** The interaction network constructed using Cytoscape visualizing the shared targets among the three datasets. **(C)** Protein-protein interaction (PPI) network of the 71 overlapping genes generated via the STRING database. **(D–F)** Gene Ontology (GO) enrichment analyses for biological process, molecular function, and cellular component terms, respectively. **(G)** KEGG pathway enrichment analysis.

Pharmacophore-based target prediction identified 667 potential targets of ALA across six databases. A total of 8,548 AMD-associated genes and 484 ferroptosis-related genes were collected from GeneCards, CTD, OMIM, and FerrDb V2, respectively. A three-way intersection analysis yielded 71 overlapping targets shared among ALA, AMD, and ferroptosis (Figure 1A). With these genes, an ALA-ferroptosis-AMD interaction network was then constructed in Cytoscape, comprising 74 nodes and 213 edges, with an average node degree of 5.757, network heterogeneity of 2.330, density of 0.079, and centrality of 0.919 (Figure 1B). Furthermore, The STRING-derived PPI network (815 edges) revealed a key iron homeostasis module (HMOX1-FTL-SLC40A1) and a redox signaling hub (MAPK1-NOS2-JUN), as well as the hub genes mediating the crosstalk between GSH metabolism and mitochondrial function (Figure 1C; Table 1).

**TABLE 1:** The top 10 hub genes with the highest degrees.

Gene name	Description	Degree
TP53	Tumor Protein P53	55
PPARG	Peroxisome Proliferator Activated Receptor Gamma	52
IL6	Interleukin 6	52
IL1B	Interleukin 1 Beta	47
JUN	Jun Proto-Oncogene, AP-1 Transcription Factor Subunit	46
NFE2L2	NFE2 Like BZIP Transcription Factor 2	45
SIRT1	Sirtuin 1	44
HMOX1	Heme Oxygenase 1	44
STAT3	Signal Transducer and Activator of Transcription 3	44
TGFB1	Transforming Growth Factor Beta 1	41

GO and KEGG functional enrichment analyses revealed a total of 2,667 enriched terms. Notably, top terms in the enriched biological processes included cellular response to metal ion, cellular response to reactive oxygen species and oxidative stress (Figure 1D). Molecular functions featured superoxide-generating NAD(P)H oxidase activity and MAP kinase activity (Figure 1E). Cellular component terms were enriched for NADPH oxidase complex and chromatin silencing complex (Figure 1F). KEGG pathway analysis highlighted the AGE-RAGE signaling pathway, the reactive oxygen species signaling pathway, the lipid and atherosclerosis pathway (Figure 1G).

### 3.2 Transcriptomic profiling and immune landscape analysis

**FIGURE 2:**
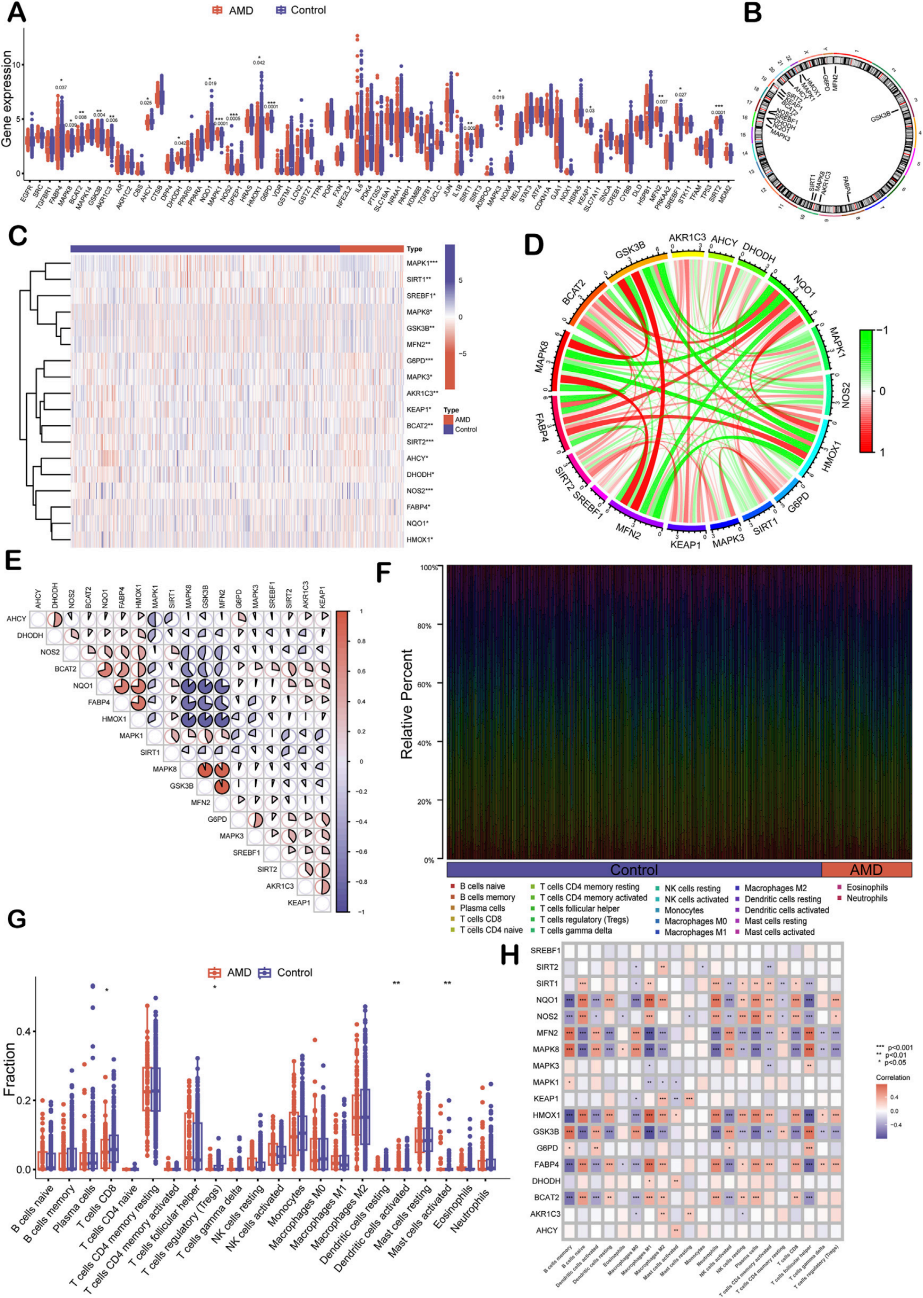
Transcriptomic validation and immune correlation analysis of core ferroptosis-related genes in AMD. **(A)** Box plots showing differential expression of 18 core genes between AMD (n = 104) and control (n = 433) retinal samples. The expression levels of FABP4 (p = 0.037), BCAT2 (p = 0.008), AKR1C3 (p = 0.006), AHCY (p = 0.025), DHODH (p = 0.042), NQO1 (p = 0.019), HMOX1 (p = 0.042), G6PD (p = 0.0001), MAPK3 (p = 0.019), KEAP1 (p = 0.03), SIRT2 (p = 0.0001), MAPK8 (p = 0.039), GSK3B (p = 0.004), MAPK1 (p = 0.0001), NOS2 (p = 0.0005), SIRT1 (p = 0.009), MFN2 (p = 0.007), and SREBF1(p = 0.027) are significantly different between the controls and the patients. **(B)** Circos plot displaying chromosomal localization of core ferroptosis-related genes. **(C)** Heatmap showing the expression patterns of the 18 core genes across AMD and control samples. **(D)** Gene-gene correlation network showing co-expression relationships among the core genes. **(E)** Pairwise correlation matrix quantifying the strength of association between individual gene pairs. **(F)** Bar graph displaying the estimated proportions of immune cell subsets. **(G)** Box plots comparing immune infiltration between the AMD and control groups. **(H)** Correlation matrix showing the association between core gene expression levels and immune cell abundance. Statistical significance: *p < 0.05, **p < 0.01, **p < 0.001.

Using the AMD transcriptomic dataset GSE135092 from the GEO database, which comprises 433 control and 104 AMD samples (Supplementary Table S2), the expression profiles of the 71 previously identified intersection genes (Supplementary Table S3) were examined. Among them, 18 core differentially expressed genes (DEGs) were identified (Figure 2A): *FABP4*, *BCAT2*, *AKR1C3*, *AHCY*, *DHODH*, *NQO1*, *HMOX1*, *G6PD*, *MAPK3*, *KEAP1*, *SIRT2* were significantly downregulated in AMD compared to the control, whereas *MAPK8*, *GSK3B*, *MAPK1*, *NOS2*, *SIRT1*, *MFN2*, *SREBF1* were upregulated (*p* < 0.05), suggesting that inflammation and lipid metabolism disorders may play critical roles in AMD development. Chromosomal mapping revealed that two key ferroptosis regulators, *KEAP1* and *HMOX1*, are located at Chr19p13 and Chr22q12, respectively (Figure 2B). Hierarchical clustering and co-expression network analysis showed high inter-gene correlation, indicating their potential cooperative roles in AMD progression (Figures 2C–E). Immunoinformatic analysis using CIBERSORT and ssGSEA revealed significant reductions in CD8^+^ T cells, regulatory T cells (Tregs), activated mast cells, and dendritic cells in the Control and AMD samples (*p* < 0.05; Figures 2F,G, Supplementary Data S1). Notably, *HMOX1* expression was positively correlated with M1 macrophage polarization (*r* = 0.596), and *MAPK8* was strongly associated with the recruitment of follicular helper T cells (*r* = 0.77, *p* < 0.001; Figure 2H). These results collectively suggest that ferroptosis-related pathways, as well as immune dysregulation, may be involved in AMD pathogenesis.

### 3.3 Machine learning-based target screening

**FIGURE 3:**
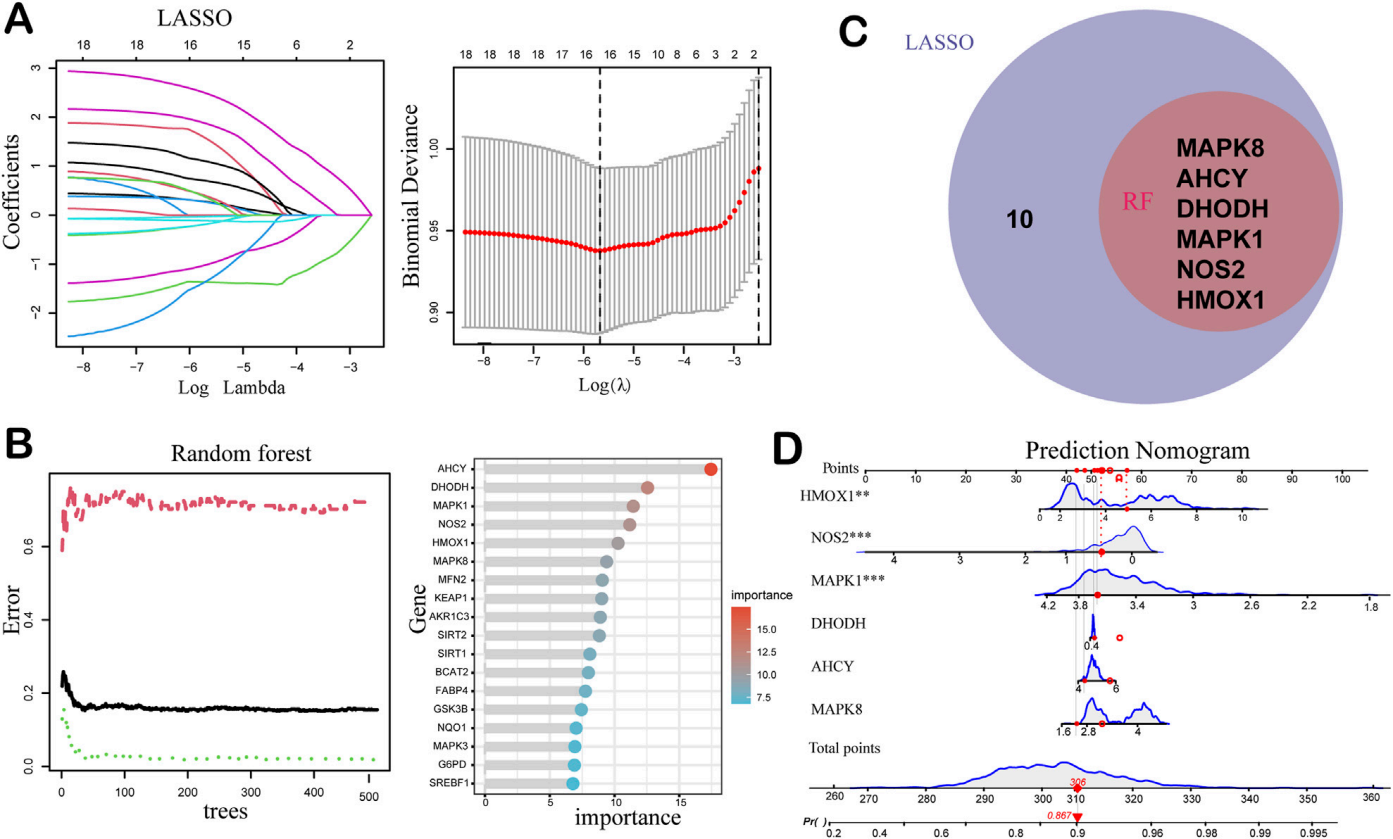
Machine learning-based identification and modeling of ferroptosis-related biomarkers for AMD. **(A)** Least Absolute Shrinkage and Selection Operator (LASSO) regression analysis of the 18 candidates using 10-fold cross-validation. In the left panel, the different colors represent the coefficients of the 16 key genes selected by machine learning. The right panel shows how the binomial deviance changes with the regularization parameter λ in the LASSO model. The red curve indicates the deviance, and the vertical gray lines represent the selected λ values. The optimal λ is typically chosen where the deviance is minimized. **(B)** Random Forest (RF) analysis ranking gene importance across 500 decision trees. In the left panel, the green, black, and red curves represent training error, out-of-bag (OOB) error, and baseline error rate (e.g., expected error from random guessing), respectively. The right panel shows the gene importance ranking in the RF model. **(C)** Venn diagram showing the intersection of LASSO and RF results, highlighting six consistently prioritized key genes. **(D)** Nomogram model integrating the expression levels of the six genes to calculate an individualized risk score for AMD progression. The total score reflects the cumulative contribution of each gene, serving as a reference for potential diagnostic or prognostic decision-making in clinical settings.

To further identify ferroptosis-related genes with high diagnostic and therapeutic potential, we employed two mainstream machine learning algorithms, LASSO regression and RF, to the 18 core differentially expressed genes (DEGs) identified in prior multi-omics analysis. Specifically, LASSO regression was used for feature compression and variable selection, yielding 16 genes with non-zero coefficients, indicating their substantial contribution to AMD risk prediction (Figure 3A; Table 2). Meanwhile, the RF classification model identified six key genes with importance values >9.0, including *AHCY*, *DHODH*, *MAPK1*, *MAPK8*, *NOS2*, and *HMOX1*, significantly exceeding those of other candidate genes (Figure 3B; Table 3). The intersection between the LASSO and RF-identified targets confirmed these six genes as overlapping top-ranked features (Figure 3C). Subsequently, a multivariate nomogram model was constructed by assigning weights to each gene’s expression level to compute sample-specific risk scores (Figure 3D), showing strong discriminatory power in the GSE135092 dataset. These six genes were subsequently prioritized for downstream molecular docking and *in vivo* validation, serving as candidate targets for exploring the therapeutic mechanism of ALA in AMD.

**TABLE 2:** List of genes identified by LASSO regression.

Gene	Description
FABP4	Fatty Acid Binding Protein 4
MAPK8	Mitogen-Activated Protein Kinase 8
BCAT2	Branched-chain-amino-acid aminotransferase, mitochondrial
AKR1C3	Aldo-keto reductase family 1 member C3
AHCY	Adenosylhomocysteinase
DHODH	Dihydroorotate Dehydrogenase (Quinone)
MAPK1	Mitogen-Activated Protein Kinase 1
NOS2	Nitric Oxide Synthase 2
MAPK3	Mitogen-Activated Protein Kinase 3
KEAP1	Kelch Like ECH Associated Protein 1
HMOX1	Heme Oxygenase 1
G6PD	Glucose-6-Phosphate Dehydrogenase
SIRT2	NAD-dependent deacetylase sirtuin-2
SIRT1	Sirtuin 1
MFN2	Mitofusin 2
SREBF1	Sterol regulatory element-binding protein 1

**TABLE 3:** List of the six key genes from intersection of LASSO and RF algorithms.

Gene	Description	Gini importance scores
MAPK8	Mitogen-Activated Protein Kinase 8	9.394
AHCY	Adenosylhomocysteinase	17.44
DHODH	Dihydroorotate Dehydrogenase	12.551
MAPK1	Mitogen-Activated Protein Kinase 1	11.446
NOS2	Nitric Oxide Synthase 2	11.171
HMOX1	Heme Oxygenase 1	10.273

### 3.4 Molecular docking and molecular dynamics (MD) simulation

**FIGURE 4:**
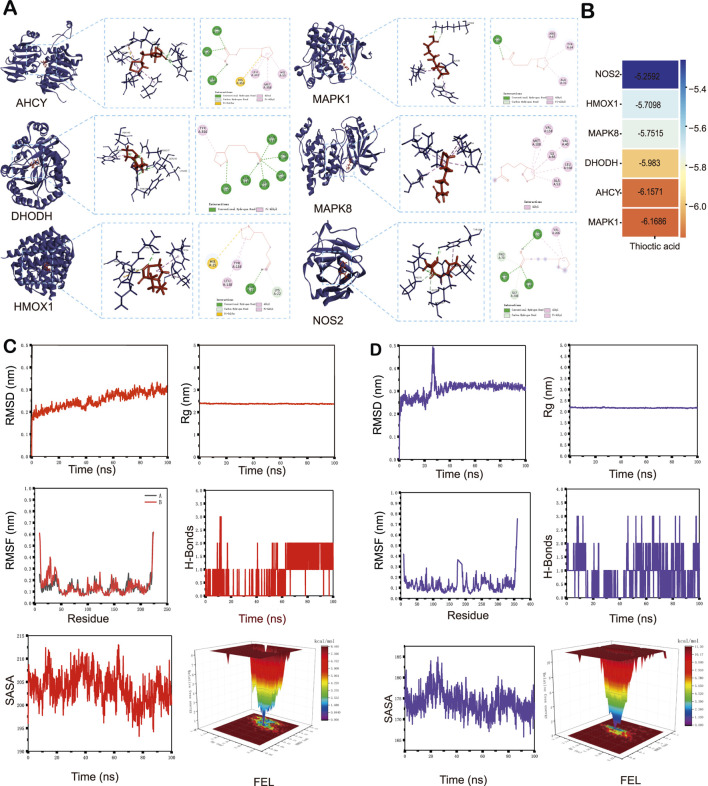
Molecular docking and molecular dynamics (MD) simulations. **(A)** Representative docking conformations showing ALA binding to six core proteins implicated in AMD-associated ferroptosis. **(B)** Heatmap summarizing the docking affinities and dissociation constants (Kd) of ALA across the six targets. **(C)** MD simulation of the HMOX1-ALA complex. **(D)** MD simulation of the MAPK1-ALA complex. RMSD, root mean square deviation. RMSF, root mean square fluctuation. Rg, Radius of gyration. H-bonds, hydrogen bonds. SASA, solvent-accessible surface area. FEL, free energy landscape.

We then performed molecular docking experiments to evaluate the binding affinity and interaction patterns of the ALA molecule to the six core molecular targets (Figure 4A). ALA showed favorable binding affinity to all six proteins at physiological temperature (37°C). Key interaction features included: hydrogen bonding between ALA and LYS-54 in MAPK1 (PDB: 4QTA); π-π stacking with His-353 in AHCY (PDB: 1LI4); binding to the ubiquinone site in DHODH (PDB: 4IGH); hydrophobic interactions with LEU-168 in MAPK8 (PDB: 4QTD); localization near the heme-binding pocket of HMOX1 (PDB: 1N45); and polar contacts involving ARG-68, TYR-120, and VAL-71 in NOS2 (PDB: 5XN3). The binding free energies ranged from −5.26 to −6.17 kcal/mol, with dissociation constants (Kd) between 28.9 and 148.3 μM (Figure 4B; Table 4).

**TABLE 4:** The binding free energies and dissociation constants between α-lipoic acid and its potential targets.

Gene	Binding free energy (kcal/mol)	Dissociation constants (μM)
MAPK8	−5.7515	66.7
AHCY	−6.1571	29.8
DHODH	−5.983	46.2
MAPK1	−6.1686	28.9
NOS2	−5.2592	148.3
HMOX1	−5.7098	73.4

Given the central role of HMOX1 in ferroptosis and the strong binding affinity observed for MAPK1, these two complexes were selected for 100-nanosecond MD simulations to assess the stability and binding behavior in detail. For HMOX1, the root mean square deviation (RMSD) stabilized between 0.21 and 0.30 nm after 45 ns (Figure 4C), while the MAPK1 complex reached stability between 0.31 and 0.36 nm after 40 ns (Figure 4D). Radius of gyration (Rg) values for HMOX1 and MAPK1 remained stable within 2.25–2.50 nm and 2.00–2.50 nm, respectively, suggesting compact and stable protein structures during simulation. Root mean square fluctuation (RMSF) profiles showed similar trends, with fluctuations remaining below 0.7 nm in both complexes, suggesting high protein rigidity and dynamic stability. Hydrogen bond analysis revealed 0–3 stable H-bonds in the HMOX1 complex, whereas the MAPK1 system exhibited more variability, reflecting greater conformational dynamics. Solvent-accessible surface area (SASA) remained relatively stable throughout the simulation process for both complexes. Finally, free energy landscape (FEL) analysis showed energy minima located in low RMSD and low Rg regions, suggesting that both complexes adopt stable conformations during the simulation.

Moreover, we evaluated and decomposed the binding free energy of the two complexes using MM-PBSA analysis (Supplementary Figure S1). For HMOX1, the gas-phase binding energy (GGAS) was −40.97 kcal/mol, with favorable van der Waals (VDWAALS <0) and electrostatic (EEL <0) contributions (Supplementary Figure S1A). The solvation energy (GSOLV) was 20.43 kcal/mol, including a small favorable non-polar term (ESURF <0) and an unfavorable polar solvation term (EGB >0). The total binding free energy, i.e., the sum of GGAS and GSOLV, was −20.54 ± 5.01 kcal/mol. The key contributing residues included HIS25, ALA28, MET34, LEU138, GLY139, and PHE207 in HMOX1 (Supplementary Figure S1B). The MAPK1-ALA complex showed a similar energy contribution pattern, with a favorable GGAS and a partially unfavorable GSOLV, leading to a total a binding free energy of −17.30 ± 6.33 kcal/mol (Supplementary Figure S1C), with dominant interactions involving residues GLU71, LEU115, LEU121, HIS125, LEU157, THR159, and CYS161 (Supplementary Figure S1D).

### 3.5 ALA attenuates SI-induced visual dysfunction

**FIGURE 5:**
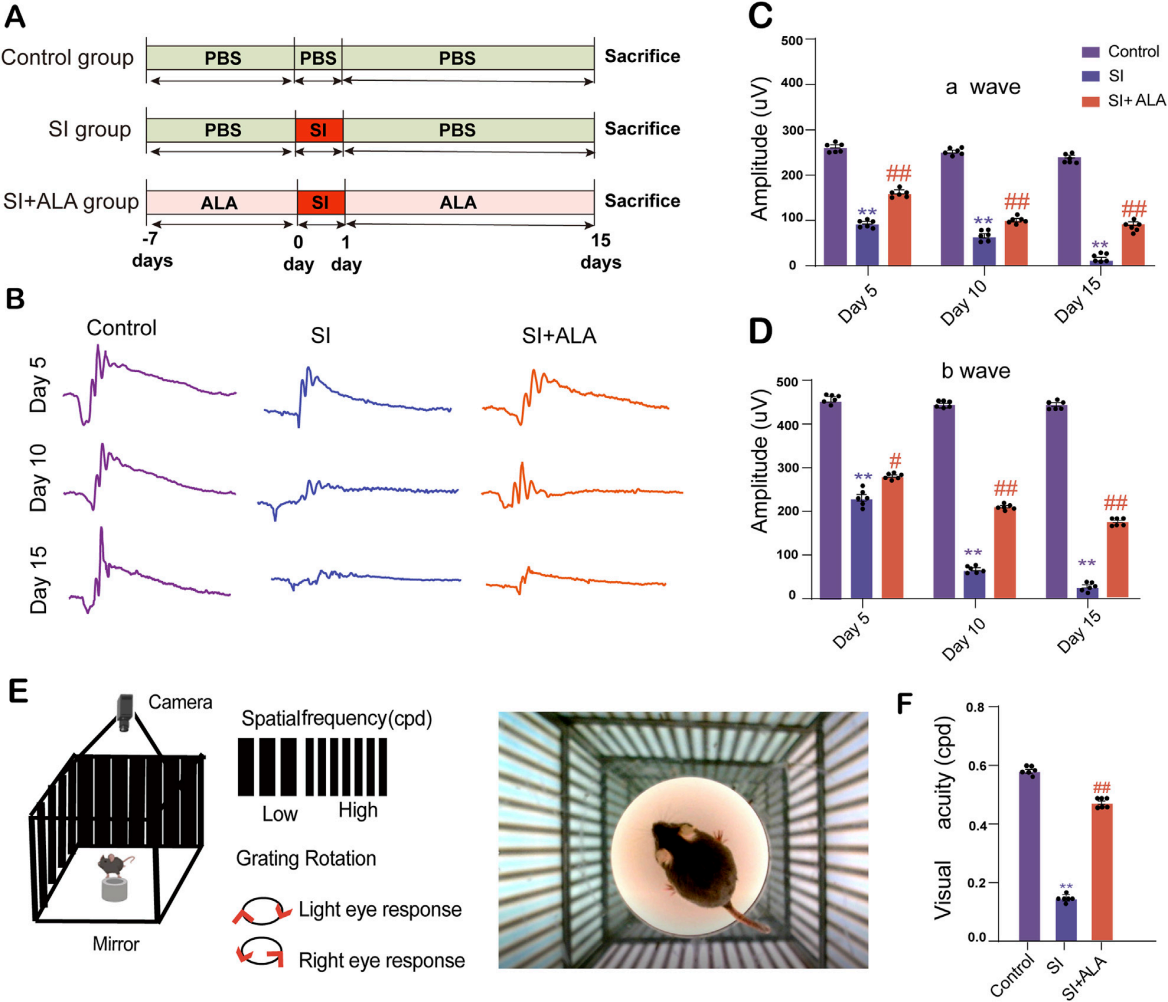
ALA preserves retinal electrophysiological function and visual acuity in a sodium iodate (SI)-induced retinopathy model. **(A)** Time course of the SI and ALA treatments. **(B)** Representative full-field electroretinogram (ERG) waveforms recorded at days 5, 10, and 15 post-SI injection in control, SI-treated, and SI + ALA-treated mice. **(C,D)** Quantification of mean a-wave and b-wave amplitudes. The a-wave reflects photoreceptor activity, while the b-wave corresponds to inner retinal responses, elicited by 3.0 cd·s/m^2^ white light flashes. **(E)** Schematic of the optomotor response (OMR) test used to assess spatial frequency thresholds. **(F)** Visual acuity measurements demonstrating SI-induced visual impairment and partial recovery with ALA treatment. Data are presented as mean ± SEM (n = 6 biologically independent mice per group). Data are presented as mean ± SEM (n = 6 biologically independent mice per group). Statistical significance determined by one-way ANOVA with Dunnett’s *post hoc* test: **p < 0.01, *p < 0.05 (SI vs. control); ##p < 0.01, #p < 0.05 (SI + ALA vs. SI).

To validate the effects of ALA *in vivo*, we established a SI-induced retinal injury mouse model and evaluated the electrophysiological and visual functions of the mice (Figure 5A). The representative full-field ERG waves of the control, SI, and SI + ALA groups are shown in Figure 5B. Quantitative analyses further showed that, compared to the control group, the a- and b-wave amplitudes were significantly reduced in the SI group in a time-dependent manner, with the most significant deficits observed on day 15 (a-wave: 7.2% of control; b-wave: 6.4% of control; p < 0.001). In contrast, ALA treatment exhibited a significant protective effect (a-wave: 46.5% of control on day 15; b-wave: 39.8% of control on day 15; p < 0.001), representing 6.5-fold and 6.2-fold improvements over the SI group, respectively (Figures 5C,D). Furthermore, the OMR assay confirmed partial preservation of visual acuity in the SI + ALA group (Figure 5E). Visual thresholds in the SI + ALA group remained at 77.1% of the control levels, compared to only 19.4% in the SI group (p < 0.001; Figure 5F), representing a ∼4.0-fold improvement. These results clearly demonstrate that ALA treatment preserves retinal electrophysiological function and visual behavior *in vivo*.

### 3.6 ALA ameliorates retinal degeneration, iron overload, and oxidative stress in SI-induced AMD mice

**FIGURE 6:**
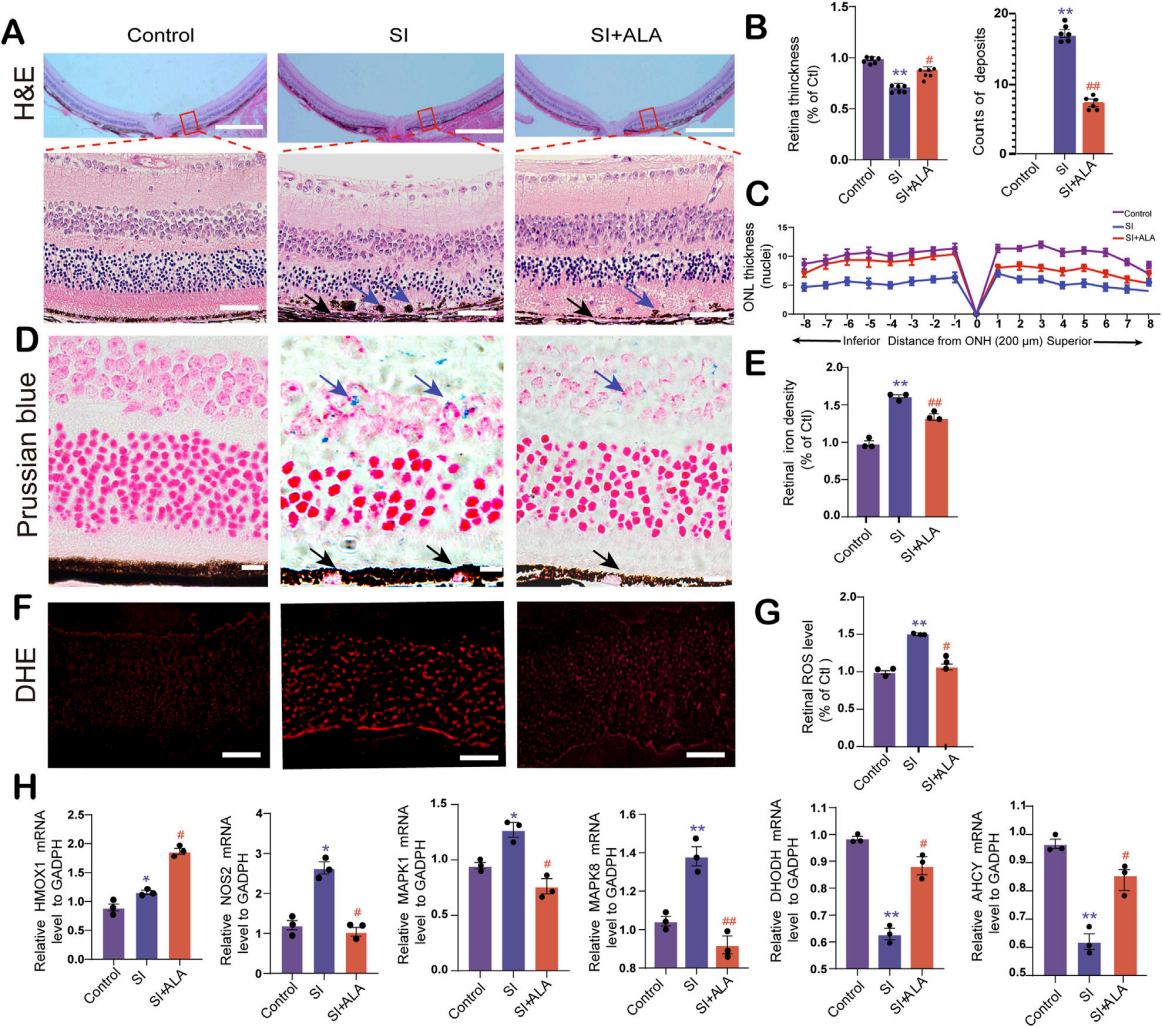
ALA attenuates SI-induced retinal degeneration, iron accumulation, oxidative stress, and ferroptosis-related gene dysregulation. **(A)** Hematoxylin and eosin (H&E) staining of retinal sections at matched eccentricities from the optic nerve head (ONH) in control, SI-treated, and SI + ALA-treated mice (upper panels). The lower panels show magnified images of the tissue within the red box. The scale bars of the upper and lower panels represent 200 and 50 μm, respectively. **(B)** Total retinal thickness (from RPE to inner limiting membrane) and counts of iron deposits in the three groups. **(C)** Spider plot of outer nuclear layer (ONL) thickness across superior and inferior retina at 200 μm intervals from the ONH. **(D)** Prussian blue staining revealing iron accumulation (blue arrows) in retinal tissues. Blue arrows indicate iron accumulation. Black arrows indicate depigmented RPE cells. Scale bar: 20 μm. **(E)** Quantification of normalized retinal iron density (normalized to controls). **(F)** Dihydroethidium (DHE) fluorescence imaging indicating superoxide levels in retinal sections. Scale bar: 50 μm. **(G)** Quantification of retinal reactive oxygen species (ROS) levels (normalized to controls). **(H)** qRT-PCR analysis of ferroptosis-related gene (HMOX1, NOS2, MAPK1, MAPK8, DHODH, and AHCY) expression levels, normalized to GAPDH. Data are presented as mean ± SEM (n = 3 biologically independent animals; 4 retinal sections per replicate). Statistical significance determined by one-way ANOVA with Dunnett’s *post hoc* test: **p < 0.01, *p < 0.05 (SI vs. control); ##p < 0.01, #p < 0.05 (SI + ALA vs. SI).

Having confirmed the protective effects of ALA in SI-induced AMD mice, we further investigated its impact at the tissue and cellular levels. Histopathological analysis revealed that retinas from SI-treated mice were significantly thinner, with disorganized cellular architecture and abnormal deposit accumulation compared to the control group (Figures 6A,B). In contrast, ALA administration reduced the number of retinal deposits and significantly improved retinal thickness compared to the SI group (*p* < 0.001). To account for potential distortion from tissue edema or intercellular fluid, we quantified the number of nuclei in the outer nuclear layer (ONL) across defined intervals (200–1,600 μm from the optic nerve head). ALA treatment effectively rescued SI-induced ONL thinning (Figure 6C).

Prussian blue staining demonstrated substantially enhanced iron accumulation in SI-treated retinas compared to the controls. Significantly more iron-positive cells (blue arrows) could be observed in the inner retina in the SI group than in the control group (*p* = 0.006, Figures 6D,E). Meanwhile, due to SI-induced pigment epithelial atrophy, more depigmented RPE cells were observed in the SI group than in the SI + ALA group (black arrows, Figure 6D). In contrast, ALA treatment significantly reduced SI-induced iron deposition in the inner retina, with a 74.9% decrease in iron-positive cells compared to the SI group (*p* = 0.029; Figures 6D,E). Contrast-enhanced images further confirmed greater iron levels in the SI group compared to the control group, which was markedly reduced following ALA treatment (Supplementary Figure S2). Furthermore, DHE fluorescence analysis demonstrated significantly elevated ROS levels in SI-treated retinas, which were reduced by 22.2% following ALA treatment (*p* < 0.001; Figures 6F,G), suggesting substantial mitigation of oxidative stress. Additionally, we measured the retinal and serum levels of malondialdehyde (MDA) and GSH of the mice (Supplementary Figure S3). The retinal and serum MDA levels were significantly elevated in the SI-treated group, while GSH levels were decreased; notably, ALA treatment significantly reduced MDA levels and restored GSH levels, suggesting that ALA treatment could effectively relieve the oxidative stress in SI-treated mice. These pieces of evidence support the role of ALA as a potent iron chelator capable of reversing SI-induced iron deposition and pigmentary disruption.

Subsequently, we explored the expression levels of the six core genes identified previously. Using qRT-PCR, we found that *Nos2* and *Mapk8* were significantly upregulated following SI treatment, along with moderate but significant increases in *Hmox1* and *Mapk1* (*p* < 0.05, Figure 6H). ALA treatment further enhanced the expression level of *Hmox1*, while suppressing those of *Nos2*, *Mapk8*, and *Mapk1*. On the contrary, following SI treatment, the expression levels of metabolic regulators, *Ahcy* and *Dhodh*, were significantly downregulated by 55% and 48%, respectively (*p* < 0.05, Figure 6H), both of which were restored by ALA treatment (*p* < 0.05). Together, these findings suggest that ALA may alleviate SI-induced retinal degeneration by reducing iron accumulation, mitigating oxidative stress, and modulating ferroptosis-related gene expression.

## 4 Discussion

In this study, we systematically investigated the therapeutic potential of ALA in AMD, focusing on its regulatory role in ferroptosis-related pathways. By integrating network pharmacology, transcriptomic profiling, and machine learning, we identified six core ferroptosis-associated genes, including *AHCY*, *DHODH*, *MAPK1*, *MAPK8*, *NOS2*, and *HMOX1*, as potential targets of ALA. Molecular docking and MD simulations demonstrated stable binding between ALA and these targets, particularly HMOX1 and MAPK1. We then used SI-induced AMD mouse model to validate the protective effects of ALA, showing that ALA significantly preserved retinal structure and function, mitigated oxidative stress and iron accumulation, and modulated the expression of the previously identified ferroptosis-related genes *in vivo*. Collectively, these findings demonstrate that ALA alleviates AMD by targeting ferroptosis-related molecular pathways, offering new insights into mechanism-based therapeutic strategies for AMD.

The ferroptosis related six core genes identified through network pharmacology play distinct roles in the pathogenesis of AMD. For example, AHCY promotes GSH biosynthesis and thereby suppresses ferroptosis (Cao et al., 2020). Consistent with this, we observed a significant reduction in AHCY mRNA expression following SI treatment, which was reversed by ALA administration. DHODH is a mitochondrial enzyme in the pyrimidine synthesis pathway and has recently been recognized as a non-canonical ferroptosis suppressor by maintaining mitochondrial redox homeostasis (Mao et al., 2021); inhibition of DHODH could induce ferroptosis in cells. This also aligns with our findings that the DHODH level was downregulated after SI treatment and restored upon ALA treatment. On the contrary, MAPK1 (ERK2) and MAPK8 (JNK1) are key effectors of the MAPK signaling cascade and are known to be upregulated in AMD and contribute to photoreceptor degeneration via modulating oxidative stress response (Kyosseva, 2016). Notably, recent study suggested that the activation of the MAPK pathway may regulate ferroptosis by influencing lipid peroxidation and redox-sensitive transcription factors (Chen et al., 2023). Our finding supports this insight as both MAPK1 and MAPK8 were upregulated in SI-induced AMD mice and were downregulated by ALA treatment. NOS2, encoding inducible nitric oxide synthase (iNOS), may contribute to mitochondrial dysfunction via excess nitric oxide (NO) and reactive nitrogen species (RNS) and plays an important role in AMD pathogenesis (Toma et al., 2021). NOS2 has also been shown as a regulator in ferroptosis but its specific role remains unclear (Zhang et al., 2023). Our findings showed that NOS2 expression followed a similar trend to MAPK1 and MAPK8 upon SI and ALA treatment. Finally, HMOX1 is known to play a dual role in ferroptosis: it is cytoprotective at moderate expression levels by mitigating oxidative stress, but becomes pro-oxidant at high expression levels due to iron accumulation, potentially leading to ferroptosis through non-canonical pathways (Suttner and Dennery, 1999; Tang et al., 2021). Furthermore, it has been shown that high levels of HMOX1 could induce retinal degeneration (Li et al., 2021). In this study, HMOX1 was slightly upregulated following SI treatment but markedly induced by ALA, a finding that warrants further mechanistic investigation.

Interestingly, despite molecular docking and MD simulations showing stable binding of ALA to all six targets, our transcriptomic and qPCR analyses revealed that ALA binding did not uniformly suppress or activate gene expression; rather, it led to upregulation of certain genes (HMOX1, AHCY, DHODH) and downregulation of others (MAPK1, MAPK8, NOS2). This observation suggests that ALA may not function merely as a classical inhibitor or agonist of its predicted targets, despite the direct interactions suggested by molecular docking or dynamics simulations. ALA and its metabolic product have been shown to directly inhibit histone deacetylases (HDACs), potentially through chelating the Zn^2+^ ion at the catalytic site (Lechner et al., 2023). However, this mechanism cannot account for its predicted interactions with targets such as MAPK1, MAPK8, or HMOX1, as these proteins do not possess metal ion-dependent active sites. This highlights the need for further experimental validation to determine whether ALA affects these targets directly or via upstream regulatory pathways. Instead, ALA may serve as a context-dependent modulator of signaling networks. ALA is known to act through redox-sensitive mechanisms, influencing upstream transcriptional regulators such as NRF2 and NF-κB (Lee and Hughes, 2002; Wu et al., 2023), which in turn can differentially regulate target genes depending on cell type, redox state, and epigenetic context. For example, Nrf2 may mediate ALA-induced HMOX1 upregulation. It has been demonstrated that ALA treatment could induce rapid Nrf2 translocation to the nucleus and enhance its binding to the antioxidant response elements (AREs) in the HMOX1 gene promoter, which in turn enhances HMOX1 expression (Ogborne et al., 2005). In parallel, ALA may reduce excessive cellular ROS and sustain mitochondrial integrity, thereby creating permissive conditions for Nrf2 activation, thus indirectly upregulating HMOX1 (Lee et al., 2019). In contrast, ALA may lead to the downregulation of MAPK1 and MAPK8 through attenuating oxidative stress by scavenging ROS and enhancing cellular antioxidant defenses, thereby suppressing ROS-dependent activation of MAPK1 and MAPK8 pathways (Choi et al., 2015). Additionally, ALA could also inhibit pro-inflammatory signaling cascades (e.g., TNF-α-induced cascades), reducing upstream kinase activation and consequently reducing the expression and phosphorylation of MAPK1 and MAPK8 (Sabio and Davis, 2014; Zhang and Frei, 2001). Overall, ALA’s antioxidant, iron-chelating, and mitochondrial-modulating activities (Salehi, et al., 2019), may lead to divergent transcriptional outcomes even among functionally related ferroptosis genes. This non-linear regulatory pattern underscores the complexity of ALA’s mechanism of action and highlights the importance of systems-level approaches in understanding small-molecule therapeutics.

On top of its diverse activities, ALA may exert therapeutic benefits in AMD through its pharmacokinetic characteristics and immunomodulatory potential. Systemic administration of ALA has been shown to penetrate both the outer and inner blood-retinal barriers, enabling effective bioavailability within retinal tissues and supporting its application as a non-invasive therapeutic agent (Liu et al., 2016; Salehi, et al., 2019). In addition, our transcriptomic and immunoinformatic analyses revealed that ferroptosis-related genes targeted by ALA were closely associated with immune cell infiltration patterns in AMD tissues. Specifically, these genes correlated with increased M1 macrophages and cytotoxic CD8^+^ T cells, alongside decreased regulatory T cells and M2 macrophages, indicating a shift toward a pro-inflammatory immune microenvironment. Given that ferroptosis itself can trigger immunogenic cell death and release of damage-associated molecular patterns (DAMPs) (Dang et al., 2022), it is plausible that ALA-mediated inhibition of ferroptosis contributes to immune rebalancing by reducing oxidative stress and dampening inflammation. These findings suggest that ALA may protect against AMD not only through direct cytoprotection, but also by restoring immune homeostasis within the retina.

Another interesting observation is that, while SI treatment resulted in comparable reductions in ONL thickness across both the superior and inferior regions of the retina, ALA treatment produced a more pronounced rescue effect in the inferior retina. A similar phenomenon was reported in a previous study using a light-induced damage model, where the superior retina was believed to have sustained more severe injury, thereby limiting the extent of therapeutic rescue (Zhao, et al., 2014). One possible explanation is that Figure 6C reflects the number of nuclei layers in the ONL rather than the overall ONL thickness, which may be influenced by local edema or tissue spacing. Anatomically, the superior retina in C57BL/6 mice is known to be enriched in rod photoreceptors with high density, whereas the inferior retina contains more cone photoreceptors. Rods are more vulnerable to oxidative stress due to their higher metabolic demands and limited repair capacity. Consequently, SI-induced damage may be more severe in the superior retina, where rods predominate. Additionally, differences in blood-retinal barrier integrity and local drug pharmacokinetics may contribute to the region-specific efficacy of ALA. Further investigation will be required to elucidate the underlying mechanisms.

Despite these promising findings, several limitations should be acknowledged. Firstly, although our study integrates advanced multi-omics analysis with experimental validation, we did not provide direct molecular evidence, such as mass spectrometry data, to confirm the binding of ALA to its predicted targets. While molecular docking and network pharmacology analyses suggested potential interactions between ALA and several key regulators in the ferroptosis pathway, these predictions remain computational and require biochemical verification. Molecular pull-down assays, activity assays, and quantitative analysis of ferroptosis-related proteins are needed to validate these interactions in both *in vitro* and *in vivo* systems. Secondly, while the SI-induced model reproduces certain key features of retinal degeneration, such as RPE loss, photoreceptor damage, and retinal thinning, it does not fully capture the complex and chronic progression of human dry AMD. Unlike humans, rodents lack the macula and fovea, and the multifactorial pathogenesis of AMD, involving aging and genetic and environmental factors, cannot be fully recapitulated in a single model (Kim and Qian, 2022; Soundara Pandi et al., 2021). Although the SI model is widely accepted for evaluating protective strategies in advanced dry AMD (Zhao, et al., 2014), its acute nature limits its ability to model the slow pathological transitions characteristic of intermediate AMD. Further investigations using different models (e.g., lipid oxidation-induced AMD mice and human cell lines) are required to understand the roles of ALA in the development and progression of AMD, as well as to elucidate the underlying molecular mechanisms. Also, we only explored a single dose in this study (10 mg/kg/day) in the mice models. Additional experiments exploring the dose-dependent effects of ALA on AMD models are required to provide further insights into its therapeutic potential. Thirdly, although we showed that ALA alters the expression of ferroptosis-related genes, the upstream regulatory mechanisms remain to be elucidated. Additional mechanisms, such as ALA’s iron-chelation activity, were not directly investigated. As iron dysregulation plays a central role in ferroptosis and AMD pathogenesis, elucidating whether and how ALA directly modulates retinal iron homeostasis will be an important focus of future studies. Further investigations into the involvement of transcriptional regulators or epigenetic modifications are also necessary to understand how ALA exerts its anti-AMD effects.

## 5 Conclusion

In summary, this study systematically investigated the therapeutic potential of ALA in AMD, focusing on its interactions with ferroptosis-related proteins. ALA exhibited favorable binding affinity with six core targets and significantly mitigated retinal degeneration, oxidative stress, and ferroptosis in an SI-induced AMD mouse model. These findings highlight ALA as a promising modulator of ferroptosis and highlights the need for further mechanistic investigations to fully elucidate its regulatory pathways and clinical potential.

## Data Availability

The original contributions presented in the study are included in the article/Supplementary Material, further inquiries can be directed to the corresponding author.
