# Supplementary material for: α-Lipoic acid mitigates age-related macular degeneration via ferroptosis: integrative multi-omics and network pharmacology
Source: Front Pharmacol. 2025 Jul 31;16:1626907. doi: 10.3389/fphar.2025.1626907 (PMC12350381; doi:10.3389/fphar.2025.1626907)
Supplement: Supplementary file 1 [file Supplementaryfile1.docx]

Supplementary Material

## Supplementary Figures


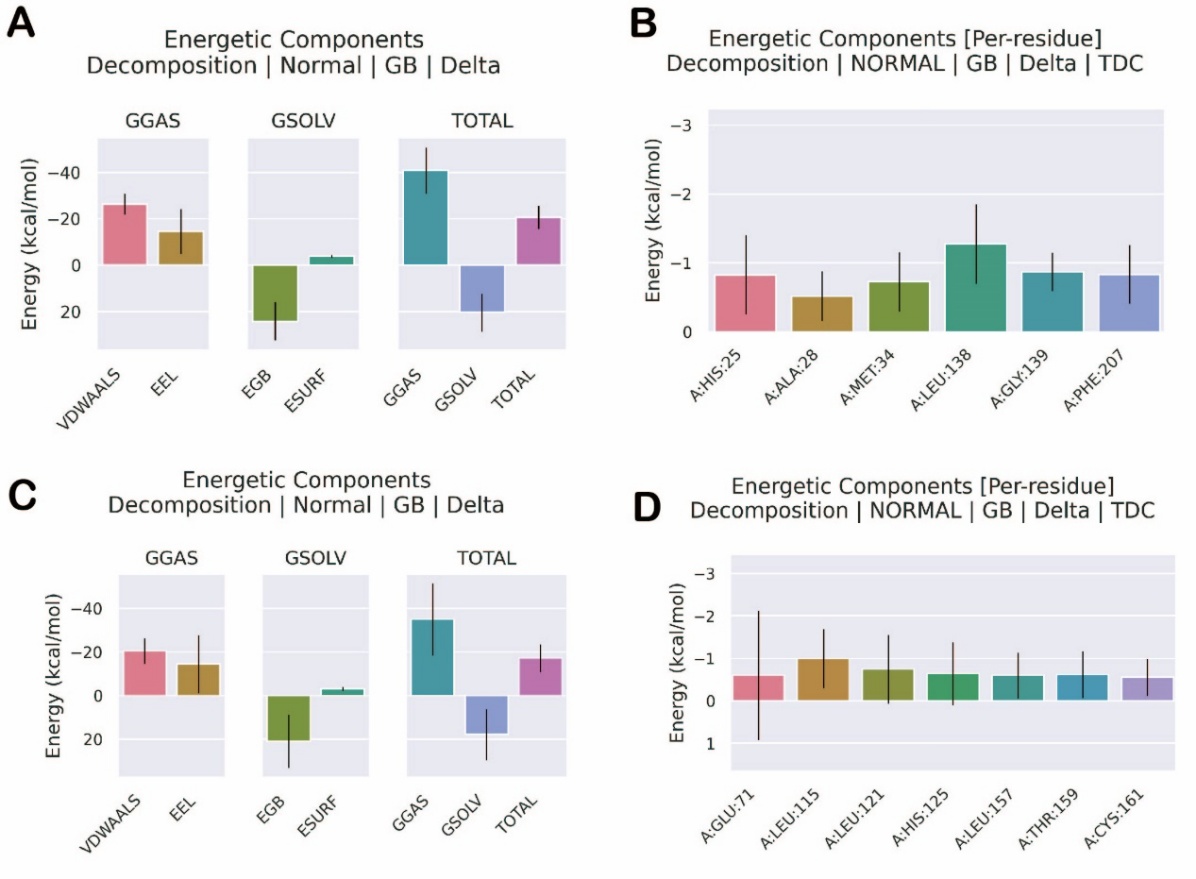


**Supplementary Fig S1. Decomposed the binding free energy of ALA-protein complexes.** (A) Gas-phase binding energy (GGAS), solvation energy (GSOLV), and total binding energy of the HMOX1-ALA complex. (B) Decomposition of the binding energy in (A) by residuals. (C) GGAS, GSOLV, and total binding energy of the MAPK1-ALA complex. (D) Decomposition of the binding energy in (C) by residuals.


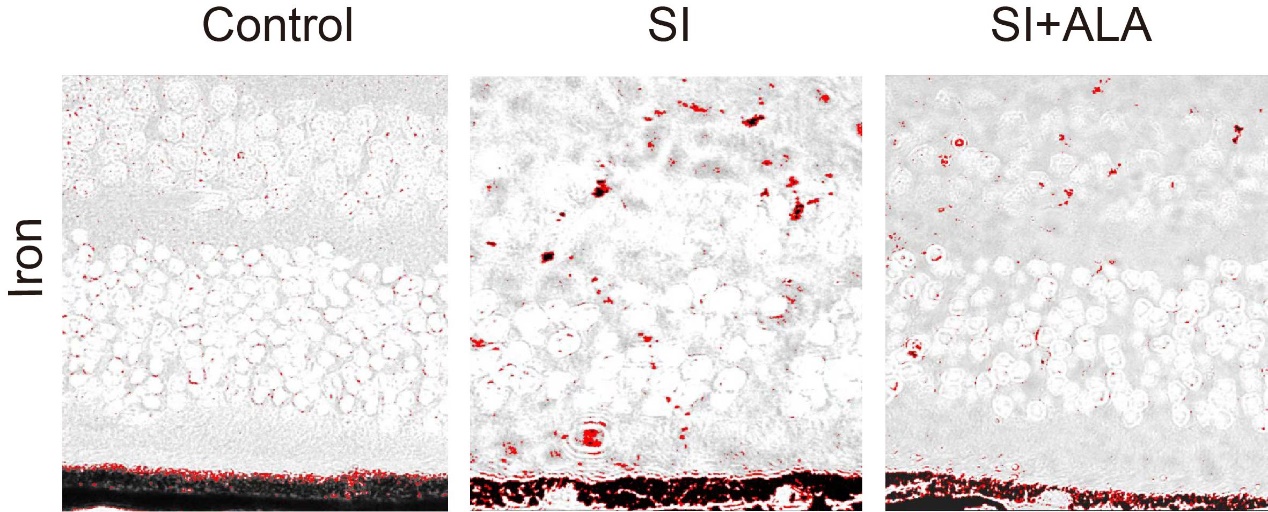


**Supplementary Fig S2.** **Contrast-enhanced images highlighting the iron levels in retina tissue (red).** The scale bar represents 20 μm.


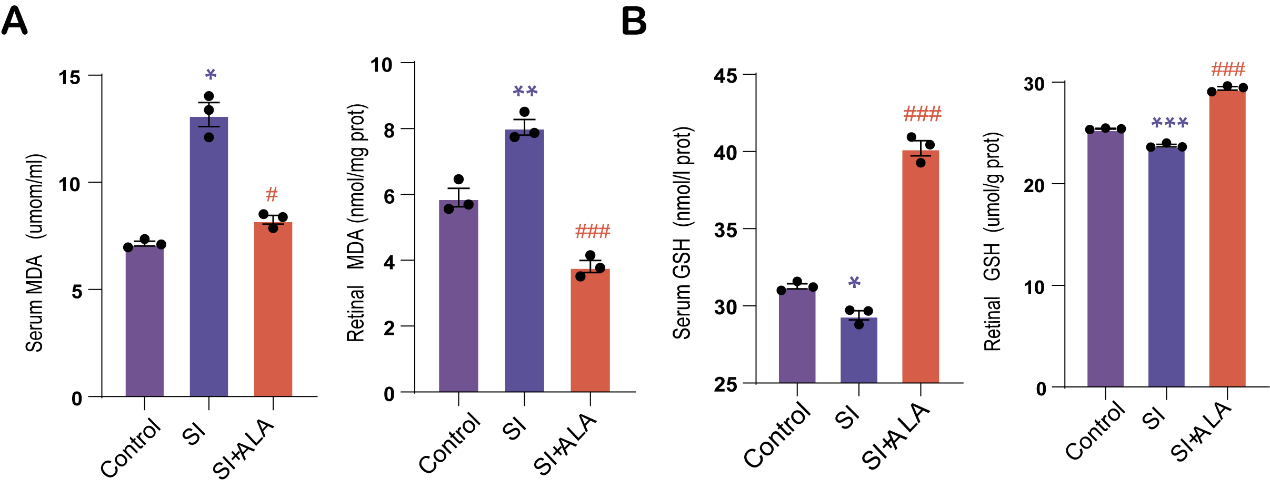


**Supplementary Fig. S3.** MDA and GSH levels in the serum and retina. (A) Serum and retinal levels of MDA in mice. (B) Serum and retinal levels of GSH in mice. All data are presented in mean ± standard error of mean (SEM) (n = 3 biologically independent animals; 4 retinal sections per replicate). **p < 0.01, *p < 0.05 (SI vs. control); ##p < 0.01, #p < 0.05 (SI+ALA vs. SI)

## Supplementary Tables

## Supplementary Table S1. Sequence of primers used for RT-qPCR analysis

| **Genes** | **Forward primer (5'-3')** | **Reverse primer (5'-3')** |
| --- | --- | --- |
| *Gapdh* | AGGTCGGTGTGAACGGATTTG | TGTAGACCATGTAGTTGAGGTCA |
| *Hmox1* | AAGCCGAGAATGCTGAGTTCA | GCCGTGTAGATATGGTACAAGGA |
| *Nos2* | TCACCTACCGCACCCGAGAT | GTACCAGGCCCAATGAGGATG |
| *Mapk8* | CTCGGAACACCTTGTCCTGAA | ACATCGGGGAACAGTTTCTCAA |
| *Dhodh* | GTTTTGTTGAGGTGGGAAGTGTG | AGCAGCATCCACCGAAGTCTTAT |
| *Mapk1* | TTCAACACACTCTATCACTGGC | AGAAGCGTTTGCGGTACTCAT |
| *Ahcy* | ATCCTTGGCCGGCACTTT | TTCTTTAGCCAGTAGCGGTCCA |

**Supplementary Table S2. Age-related macular degeneration Datasets Information list.**

| GSE135092 |  | |
| --- | --- | --- |
| Platform | | GPL16791 |
| Species | | Homo sapiens |
| Experiment type | | Expression profiling by high throughput sequencing |
| Tissue | | Retina |
| Samples in Control group | | 433 |
| Samples in AMD group | | 104 |
| Reference | | - Integration of eQTL and a Single-Cell Atlas in the Human Eye Identifies Causal Genes for Age-Related Macular Degeneration. |

**Supplementary Table S3. List of the 71 overlapping targets.**

| **Gene** | **Description** |
| --- | --- |
| EGFR | Epidermal Growth Factor Receptor |
| SRC | SRC Proto-Oncogene, Non-Receptor Tyrosine Kinase |
| TGFBR1 | Transforming Growth Factor Beta Receptor 1 |
| FABP4 | Fatty Acid Binding Protein 4 |
| MAPK8 | Mitogen-Activated Protein Kinase 8 |
| BCAT2 | Branched-chain-amino-acid aminotransferase, mitochondrial |
| MAPK14 | Mitogen-Activated Protein Kinase 14 |
| GSK3B | Glycogen Synthase Kinase 3 Beta |
| AKR1C3 | Aldo-keto reductase family 1 member C3 |
| AR | Androgen Receptor |
| AKR1C2 | Aldo-keto reductase family 1 member C2 |
| CBS | Cystathionine Beta-Synthase |
| AHCY | Adenosylhomocysteinase |
| CTSB | Cathepsin B |
| DPP4 | Dipeptidyl Peptidase 4 |
| DHODH | Dihydroorotate Dehydrogenase (Quinone) |
| PPARG | Peroxisome Proliferator Activated Receptor Gamma |
| PPARA | Peroxisome Proliferator Activated Receptor Alpha |
| NQO1 | NAD(P)H Quinone Dehydrogenase 1 |
| MAPK1 | Mitogen-Activated Protein Kinase 1 |
| NOS2 | Nitric Oxide Synthase 2 |
| DPEP1 | Beta-lactamase |
| HRAS | HRas Proto-Oncogene, GTPase |
| HMOX1 | Heme Oxygenase 1 |
| G6PD | Glucose-6-Phosphate Dehydrogenase |
| VDR | Vitamin D3 receptor |
| GSTM1 | Glutathione S-Transferase Mu 1 |
| LCN2 | Lipocalin 2 |
| GSTZ1 | Glutathione S-Transferase Zeta 1 |
| TTPA | Alpha Tocopherol Transfer Protein |
| POR | NADPH--cytochrome P450 reductase |
| FXN | Frataxin |
| NFE2L2 | NFE2 Like BZIP Transcription Factor 2 |
| IL6 | Interleukin 6 |
| PDK4 | [Pyruvate dehydrogenase (acetyl-transferring)] kinase isozyme 4, mitochondrial |
| PTGS2 | Prostaglandin-Endoperoxide Synthase 2 |
| SLC16A1 | Solute Carrier Family 16 Member 1 |
| NR4A1 | Nuclear receptor subfamily 4immunitygroup A member 1 |
| PARP1 | Poly (ADP-Ribose) Polymerase 1 |
| KDM6B | Lysine-specific demethylase 6B |
| TGFB1 | Transforming Growth Factor Beta 1 |
| GCLC | Glutamate-Cysteine Ligase Catalytic Subunit |
| JUN | Jun Proto-Oncogene, AP-1 Transcription Factor Subunit |
| IL1B | Interleukin 1 Beta |
| SIRT1 | Sirtuin 1 |
| SIRT3 | Sirtuin 3 |
| ADIPOQ | Adiponectin, C1Q And Collagen Domain Containing |
| MAPK3 | Mitogen-Activated Protein Kinase 3 |
| NOX4 | NADPH Oxidase 4 |
| RELA | Transcription factor p65 |
| STAT3 | Signal Transducer and Activator Of Transcription 3 |
| ATF4 | Activating Transcription Factor 4 |
| CDKN1A | Cyclin Dependent Kinase Inhibitor 1A |
| GJA1 | Gap Junction Protein Alpha 1 |
| NOX1 | NADPH Oxidase 1 |
| HSPA5 | Heat Shock Protein Family A (Hsp70) Member 5 |
| KEAP1 | Kelch Like ECH Associated Protein 1 |
| SLC7A11 | Cystine/glutamate transporter |
| SNCA | Synuclein Alpha |
| CREB1 | CAMP Responsive Element Binding Protein 1 |
| CYBB | Cytochrome B-245 Beta Chain |
| DLD | Dihydrolipoyl dehydrogenase |
| HSPB1 | Heat Shock Protein Family B (Small) Member 1 |
| MFN2 | Mitofusin 2 |
| PRKAA2 | Protein Kinase AMP-Activated Catalytic Subunit Alpha 2 |
| SREBF1 | Sterol regulatory element-binding protein 1 |
| STK11 | Serine/Threonine Kinase 11 |
| TFAM | Transcription Factor A, Mitochondrial |
| TP53 | Tumor Protein P53 |
| SIRT2 | NAD-dependent deacetylase sirtuin-2 |
| MDM2 | MDM2 Proto-Oncogene |
